# The where and when of COVID-19: Using ecological and Twitter-based assessments to examine impacts in a temporal and community context

**DOI:** 10.1371/journal.pone.0264280

**Published:** 2022-02-23

**Authors:** Giancarlo Pasquini, Giselle Ferguson, Isabella Bouklas, Huy Vu, Mohammadzaman Zamani, Ruixue Zhaoyang, Karra D. Harrington, Nelson A. Roque, Jacqueline Mogle, H. Andrew Schwartz, Stacey B. Scott

**Affiliations:** 1 Department of Psychology, Stony Brook University, Stony Brook, New York, United States of America; 2 Department of Computer Science, Stony Brook University, Stony Brook, New York, United States of America; 3 Center for Healthy Aging, Pennsylvania State University, University Park, Pennsylvania, United States of America; 4 Department of Psychology, University of Central Florida, Orlando, Florida, United States of America; 5 Edna Bennett Pierce Prevention Research Center, College of Health and Human Development, Pennsylvania State University, University Park, Pennsylvania, United States of America; University of Pisa, ITALY

## Abstract

In March 2020, residents of the Bronx, New York experienced one of the first significant community COVID-19 outbreaks in the United States. Focusing on intensive longitudinal data from 78 Bronx-based older adults, we used a multi-method approach to (1) examine 2019 to early pandemic (February-June 2020) changes in momentary psychological well-being of Einstein Aging Study (EAS) participants and (2) to contextualize these changes with community distress scores collected from public Twitter posts posted in Bronx County. We found increases in mean loneliness from 2019 to 2020; and participants that were higher in neuroticism had greater increases in thought unpleasantness and feeling depressed. Twitter-based Bronx community scores of anxiety, depressivity, and negatively-valenced affect showed elevated levels in 2020 weeks relative to 2019. Integration of EAS participant data and community data showed week-to-week fluctuations across 2019 and 2020. Results highlight how community-level data can characterize a rapidly changing environment to supplement individual-level data at no additional burden to individual participants.

## Introduction

Since the onset of the COVID-19 pandemic, individuals and communities have had to adapt to the evolving public health threat. Existing literature has observed increased psychological distress, anxiety, and negative affect among individuals across the pandemic period overall [1–7]. However, less is known about how individuals and communities responded across the early months of 2020, during which changes in policies, threat levels, and disease information were frequent. Leveraging longitudinal data from individuals and their surrounding community before and during the early pandemic, the present study examined changes in psychological well-being among a sample of older adults living in the Bronx, New York. Thus, the individuals in the sample represent a population of interest due to their age, geographic location, and timing of data collection, factors relevant to the study of psychological well-being during the COVID-19 pandemic.

We investigated these changes in psychological well-being from two perspectives: within-person change from a 2019 pre-pandemic baseline to 2020 during the early spike in cases in the New York City area, as well as community-level change over this period in the same area. For this study, the community was defined as a geographic community, specifically Bronx County. To examine within-person change, we used data from the Einstein Aging Study (EAS), an ongoing longitudinal measurement burst study [8]. The EAS sample consists of older adults residing in Bronx County, New York. EAS participants reported on their thoughts, mood, stress, affect, and loneliness multiple times per day for several weeks; these intensive periods of repeated surveys are referred to as measurement “bursts” of ecological momentary assessment (EMA). We compared each participant’s reports from the early pandemic (February-June 2020) to their own reports from their prior year’s measurement burst. To examine community-level change in EAS participants’ surrounding environments, we used language-based assessments [9] of community distress from public Twitter posts made by Bronx County users during the same periods as EAS data collection. We then compared the individual-level and community-level data to examine how indicators of psychological well-being from two sources correlated and fluctuated across 2019 and 2020.

Although some studies have observed a general decline in psychological well-being over the pandemic period, recent research suggests that older adults may be somewhat protected against pandemic-related distress [10,11], a finding consistent with broad interpretations of Socioemotional Selectivity Theory (SST) which posits that older adults are more likely to be motivated to maintain positive emotions and to engage in emotion regulation strategies relative to younger adults, thus promoting affective well-being [12]. According to Strength and Vulnerability Integration (SAVI) [13], however, especially when encountering stressors, pre-existing individual differences may undermine older adults’ strengths at emotion regulation and diminish age-benefits in emotional well-being compared to younger people [14]. For example, for those older adults who appraised the pandemic as challenging (perhaps due to an elevated health risk, cognitive impairment, or personality-related tendencies toward negative appraisal) may not have experienced affective protective effects of older age in the pandemic [15].

To capture this possible heterogeneity in older adults’ psychological well-being during the early pandemic, personality traits and mild cognitive impairment (MCI) measured prior to the pandemic were examined as potential moderators. Extraversion and neuroticism were selected because of their relevance to psychological well-being and aging [16,17]. MCI was tested as a moderator because participants with MCI may report worse psychological well-being, relative to participants without MCI, due to expected, non-pandemic trends (e.g., declining cognitive health across time) [18]. In the context of SAVI, MCI represents a negative experience that could contribute to lower levels of psychological well-being, independent of the pandemic.

Because the pandemic is an unfolding event with varying community public health restrictions, positivity rates, mortality rates, and individuals’ understanding of risk, making conclusions from longitudinal data can be complicated. Real-time alignment with community context is needed to understand such a dynamic event, but few data lend themselves to quantitative study at a large scale. Identifying the “start” of an unfolding event in a community can be difficult, and in the absence of an external gauge, any pre-pandemic to during-pandemic change detected could be due to numerous sources (e.g., seasonal variation, unrelated events, aging-related change). Thus, to “triangulate” [19] the likely explanation for any change observed, we incorporated longitudinal contextual data on well-being from the community in which participants resided.

To construct well-being scores representing the surrounding Bronx community in which EAS participants resided, we extracted features from public Twitter posts made by Bronx County users during the time periods when EAS participants completed their measurement bursts. The Twitter posts that were assessed were not composed by EAS participants, but rather Twitter users in Bronx County during the time of EAS data collection. Past work has shown that Twitter users can be reliable “canaries in the coal mine” for community well-being [20,21]. For example, psychological characteristics (e.g., anger, interpersonal tension) derived from language in Twitter posts within a community have been used to predict heart disease mortality risk within the same community [20]. Impressively, these Twitter extractions outperformed well-known risks from demographic, socioeconomic, and health characteristics such as smoking, diabetes, and hypertension. Thus, our second goal was to examine within-community change in well-being over the same 2019–2020 period as examined in the EAS individuals. Importantly, we investigated not only the longitudinal trends but also sought to illuminate the topics that may have produced the changes observed.

Lastly, we compared the individual and community. This novel approach of contextualizing individual-level EMA reports with Twitter community markers is uniquely suited to address questions about psychological well-being during a rapidly evolving community-level event. During the early months of 2020, COVID-19 information and risks were rapidly changing, but the way that these larger community changes impacted individuals is unknown. Given the many ways individuals interact with their communities, for psychological and other health researchers understanding the participants’ community environment may provide available, no-burden, and invaluable, but overlooked data on the impact of a shared threat.

## Methods

### Participants and data sources

#### Individual-level data

EAS participants were recruited through systematic random sampling from Medicare and New York City Registered Voter Lists for Bronx County [22]. Collection of EMA measurement burst data began in May 2017, continued throughout 2020, and is ongoing. The Albert Einstein College of Medicine Institutional Review Board (IRB) approved the study and participants provided written informed consent.

EAS follows more than 300 participants with follow-up scheduled across the calendar year. To provide the clearest examination of possible initial pandemic effects, this analysis focused on 78 EAS participants who completed a 14-day EMA burst prior to and during the initial COVID-19 outbreak (i.e., participants that provided data at two bursts). Table 1 summarizes participants’ MCI status, age, and other demographic variables reported prior to the pandemic. For this analysis, we considered participants that began their 2020 burst on or after February 1, 2020 to have completed the burst during the COVID-19 pandemic. This date roughly corresponds to when the World Health Organization declared COVID-19 a public health emergency on January 30, 2020 [23] and when the United States declared COVID-19 a public health emergency for the United States on February 3, 2020 [24]. As of February 4, 2020, cases of COVID-19 had been reported in 24 countries, including 11 confirmed cases of COVID-19 in the United States [25]. The earliest participant to complete their burst during the pandemic period began the burst on February 5, 2020. These 78 participants completed their pre-COVID burst between November 28, 2018 and October 22, 2019 with a mean of 357 days between the two bursts (see Table 1). S1 Fig in the Supplemental Material shows weekly counts of the number of participants that provided EMA data in 2019 and 2020.

**Table 1:** EAS individual difference measures.

	Mean (SD) Range/n (%)
**Personality**	
Extraversion	3.30 (.60) 2.13–4.88
Neuroticism	2.38 (.68) 1.00–4.50
**Age**	78.05 (5.58) 72–95
**Years of education**	14.78 (3.51) 2–22
**MCI**	
No	55 (70.51)
Yes	23 (29.49)
**Gender**	
Female	52 (66.67)
Male	26 (33.33)
**Race/Ethnicity**	
White ^a^	33 (42.31)
African American	34 (43.59)
Hispanic, White	10 (12.82)
Hispanic, Black	0
Asian	1 (1.28)
**Married**	
Not married	51 (65.38)
Married	27 (34.62)
**Currently working**	
Not working	73 (93.59)
Working	5 (6.41)
**Income**	
Less than $15,000	9 (11.84)
$15,000–30,000	23 (30.26)
More than $30,000	44 (57.89)
Refused/did not know	2
**Burst completed**	
1st	40 (51.28)
2nd	29 (37.18)
3rd	9 (11.54)
**Days between 2019 and 2020 bursts**	357.12 (34.57) 229–491

*Note*. EAS = Einstein Aging Study. MCI = Mild Cognitive Impairment. Individual difference variables were measured in 2019. One participant did not complete the personality measures. Burst completed represents the number of repeated measurement bursts the participant had completed including the 2019 data collection. One participant completed their pre-pandemic burst in December 2018.

^a^ The outdated and inappropriate term Caucasian was used in the EAS demographic questionnaire in 2019, but has been changed to white in this table. The EAS team and authors are revising this demographic questionnaire to more inconclusive and parallel response options, based on guidance from the literature, experts, and participants.

#### Community-level data

Language based assessments were conducted on publicly available Twitter posts from the 2019 and 2020 weeks that overlapped with EAS data collection. These posts were created by users whose IDs were mapped to Bronx County following a previously validated approach [26]. This study aimed to examine well-being across individuals and their geographic community, thus we selected Tweets from accounts that had previously been mapped to Bronx County [27] because the EAS participants were sampled from Bronx County. The mapping approach detailed in past work [26,27] was done in one of two ways. Some Tweets are geotagged to a specific location when posted because a small percentage of Twitter users have enabled geolocation coordinates on their account (this represented approximately 10% of the Tweets). Alternatively, because most Tweets did not include a geolocation, we used the free response location field where users could attach a location to each of their Tweets. Text in this field was matched to lists of unique city and location names that were within Bronx County. A subsample of Tweets that had both location information in the text field and geolocation data was used to confirm that the checking process was valid for locations in Bronx County. This automatic approach for determining the location of Tweets has previously been shown to be 93% accurate in reflecting a user’s location [26]. These methods comply with Twitter’s Terms and Conditions for the use of publicly available Tweets.

Retweets and messages containing URLs were excluded because these contain non-original content. The relative frequencies of words and phrases were then extracted and input to artificial intelligence-based models of constructs detailed below. This approach used validated algorithms which extract features (i.e., quantitative variables) from the language patterns, and then find reliable associations between combinations of features and established self-report scales, such as personality measures of the construct [28–30].

Although EAS participants were not necessarily Twitter users, the posts provide information on the conversations occurring within the Bronx community in which the EAS participants resided, during the same periods when participants were completing their EMA bursts. Twitter was selected as the social media platform for three reasons. First, past work has identified COVID-19 related topics among Tweets [31]. Second, Twitter has been used as a tool in past public health work [32], including during the H1N1 pandemic in 2009 [33] and a cholera outbreak in Haiti in 2010 [34]. Although demographic data were not collected for Twitter users, these users were likely to differ demographically compared to EAS participants (particularly on age) based on national averages of Twitter users [35]. The use of Twitter is not intended to be representative of older adults’ experiences, rather these data are meant to characterize the broader Bronx community. Past research has shown that despite the overrepresentation of younger individuals, Twitter content covaries with psychological and health outcomes in the community [20,26]. Third, as these past studies mention, Twitter’s role as a micro-blogging social media site makes it a likely platform where users would discuss the rapid changes and implications of the COVID-19 pandemic and related mitigation policies.

### Measures

#### Individual level: EAS participant data

*Individual difference measures*. Individual difference measures used in this analysis were assessed in 2019. **Personality.** Participants completed the Big Five Inventory [36]. Eight items each were used to assess extraversion and neuroticism; scores for extraversion and neuroticism were calculated as the mean of their respective items. In this sample, Cronbach’s alpha was 0.62 for extraversion and 0.71 for neuroticism. **Mild Cognitive Impairment.** MCI status was determined on the basis of comprehensive neuropsychological assessment according to Jak/Bondi criteria [22,37,38].

*Repeated measures*. EMA data used in this study were collected before and during the pandemic for each participant. **COVID Onset** was a dummy variable created to represent the time of EMA data collection with 0 representing the EMA period participants completed prior to the pandemic and 1 representing the EMA period participants completed during the pandemic.

Participants used visual analogue slider scales (scored from 0–100) on study-provided smartphones to report on each EMA item. Thought unpleasantness, subjective stress, and mood valence were assessed up to six times daily and momentary emotional states and thoughts were measured up to five times daily. **Thought Unpleasantness** was assessed with the question “In the past 5 minutes, what type of thoughts were you having?” using a slider from “unpleasant” to “pleasant”. For interpretation, this item was reverse-scored such that higher scores indicated more unpleasant thoughts. **Stress** was assessed via the question, “Right now, how stressed are you?” and used a slider from “not at all” to “extremely”. **Mood valence** was assessed with the question, “Right now how is your overall mood?” with a slider from “bad” to “good” such that higher scores represented more positive valence. Momentary feelings of **Tense/Anxious**, **Depressed**, **Frustrated**, and **Lonely** were assessed as separate items, “Right now, do you feel…?” using sliders from “not at all” to “extremely” for each item. **Thought Control and Worry** were assessed with the questions, “In the past 5 minutes were you having a train of thought that you couldn’t get out of your head?” and “In the past 5 minutes, were you thinking about personal problems or worries?,” respectively, using sliders from “not at all” to “very much”.

#### Community-level: Bronx community markers extracted from Twitter

*Depressivity and anxiety*. Artificial intelligence-based assessments of community depressivity and anxiety were extracted from Twitter posts using existing models. These models were trained in prior work by analyzing individuals’ social media posts with their responses to a validated conventional personality measure—the NEO-PI-R which includes depression and anxiety facet scores [39]. Specifically, English-speaking Facebook users (predominantly from the United States, Canada, and the United Kingdom) opted-in to complete the NEO-PI-R and provided access to their Facebook posts such that online word usage and personality traits could be correlated [28]. The NEO-PI-R assessment of anxiety and depressivity facets were extracted from Twitter posts in the current study. Scores of depressivity represent the use of negative low arousal language and scores of anxiety represent the use of negative high arousal language [30]. In these models, a machine learning-based regression model was applied to words and phrases used in Twitter posts to extract scores on the depressivity and anxiety facets at the community level [40]. Using these validated pre-trained models, continuous community scores were produced such that higher scores represent greater amounts of depressivity and anxiety. This approach has recently been used to predict psychological well-being. Specifically, depressivity and anxiety scores derived from interview transcripts using the same artificial intelligence models predicted posttraumatic stress disorder symptoms in World Trade Center attacks first responders [30].

*Affective valence*. Similar to the method used for depressivity and anxiety scores, words and phrases from Twitter posts were used to identify affective valence of language used. The model used to extract affective valence from Twitter posts in the present study was trained on a separate dataset of social media posts. In this prior work, the valence and arousal of 2,895 posts were rated by two independent psychologists (kappa > 0.80) [40]. These ratings were then used to develop a model that predicted the valence rating of posts based on the language used [41]. Applied to the present study, the frequency of words and phrases used in posts were weighted and a continuous community affective valence score of the words and phrases was produced [41]. Lower scores represent more negatively-valenced affect.

*COVID-19 topics*. Latent Dirichlet Allocation **(**LDA) method was used to identify topics based on automatically grouped clusters of words [42]. This approach was used to quantify the discussion around COVID-19 each week. Prior work derived 120 “content-specific topics” over a corpus of COVID-19-related tweets from January through August 2020 [31]. To find the topics most indicative of each week, we scored their relative frequency of the week versus all other weeks using the *log-odds IDP* metric [43]. We then summarized the topics scoring highest for the given week by plotting its most frequent words in a word cloud, where size indicates prevalence.

### Analytic plan

The individual-level data from EAS participants were analyzed using separate multi-level models for each EMA outcome using proc mixed in SAS version 9.4 [44]. Each outcome was assessed separately as opposed to calculating composite scores (e.g., a single negative affect score or a negative cognition score) as the outcome to maintain granularity. It is not clear how the pandemic may have impacted specific aspects of negative affect or negative cognition among older adults; thus, each outcome was assessed separately. In each model, COVID onset was a level 1 predictor; gender, age, MCI status, extraversion, and neuroticism were level 2 predictors. Follow-up models included COVID onset X extraversion, COVID onset X neuroticism, and the COVID onset X MCI status interaction terms.

Community markers extracted from Twitter were used to contextualize participants’ responses to EMA measures and provide descriptive information about the time during which participants were completing these EMA bursts. The correlation between Twitter community markers and corresponding EMA responses for each week was calculated for 2019 and 2020. That is, responses on the tense/anxious, depressed, and mood valence EMA items were averaged within each participant across all observations the individual completed during each week. These weekly person averages were then averaged to provide a weekly sample-average score of tense/anxious, depressed, and mood valence for each week in 2019 and 2020 (for which data were available, 32 weeks in 2019 and 19 weeks in 2020). This approach mirrors the aggregation approach used to collect the Twitter community markers such that scores of anxiety, depressivity, and affect from users’ tweets were aggregated across the week and then a mean community marker score was calculated across all users that contributed tweets during each week of the year. The aggregation approaches in the two datasets allowed for a comparison at a weekly level of how EAS participants’ individual-level reports aligned with Twitter-based community marker scores in the same week.

## Results

### Descriptive statistics

Summary statistics for the 2019 and 2020 individual-level data from EAS participants are provided in Table 2. S1 Fig in the Supplemental Material shows weekly counts of EMA participants and Twitter users. In longitudinal models for the individual-level data, likelihood ratio tests provided evidence that including the random effect of COVID onset (i.e., allowing for individual differences in slopes) improved model fit for each EMA outcome compared to models that did not include this random effect. This implies significant individual differences in the patterns of change from 2019 to 2020. All multilevel models with individual-level data used an unstructured covariance structure.

**Table 2:** Descriptive statistics of EMA measures.

	2019			2020			
	Mean	SD	Range	Mean	SD	Range	ICC
Thought unpleasantness	24.61	13.96	0.67–45.40	25.68	15.16	0.05–56.92	0.49
Thought control	17.11	14.52	0.05–59.39	19.34	19.04	0.01–73.77	0.54
Thought worry	22.95	16.24	0.32–69.27	23.08	19.61	0.19–85.42	0.51
Stress	19.67	15.46	0.25–67.14	20.11	17.34	0.11–67.50	0.49
Tense/Anxious	18.93	15.55	0.18–68.44	20.07	17.24	0.00–60.25	0.52
Lonely	13.45	16.13	0.09–94.72	17.60	20.52	0.04–99.25	0.67
Depressed	12.69	12.41	0.01–49.99	14.91	16.13	0.00–77.43	0.57
Frustrated	18.45	15.83	0.23–67.44	19.23	17.98	0.14–82.74	0.50
Mood valence	82.25	12.73	53.09–99.70	81.42	13.82	48.60–100	0.45

*Note*. EMA = Ecological Momentary Assessment. SD = Standard deviation. ICC = Intraclass correlation. ICC values were calculated using 2019 and 2020 observations. Mean values represent the sample means of the person-averages for the 2019 and 2020 bursts. Standard deviation and range values are also calculated from person-averages for each variable.

### Longitudinal individual-level psychological well-being

Separate multilevel models were used to test within-person change from 2019 to 2020 in EAS participants’ two-week average momentary ratings of thought unpleasantness, stress, mood valence, thought control, worry, feeling tense/anxious, feeling depressed, feeling frustrated, and feeling lonely.

**Fig 1:**
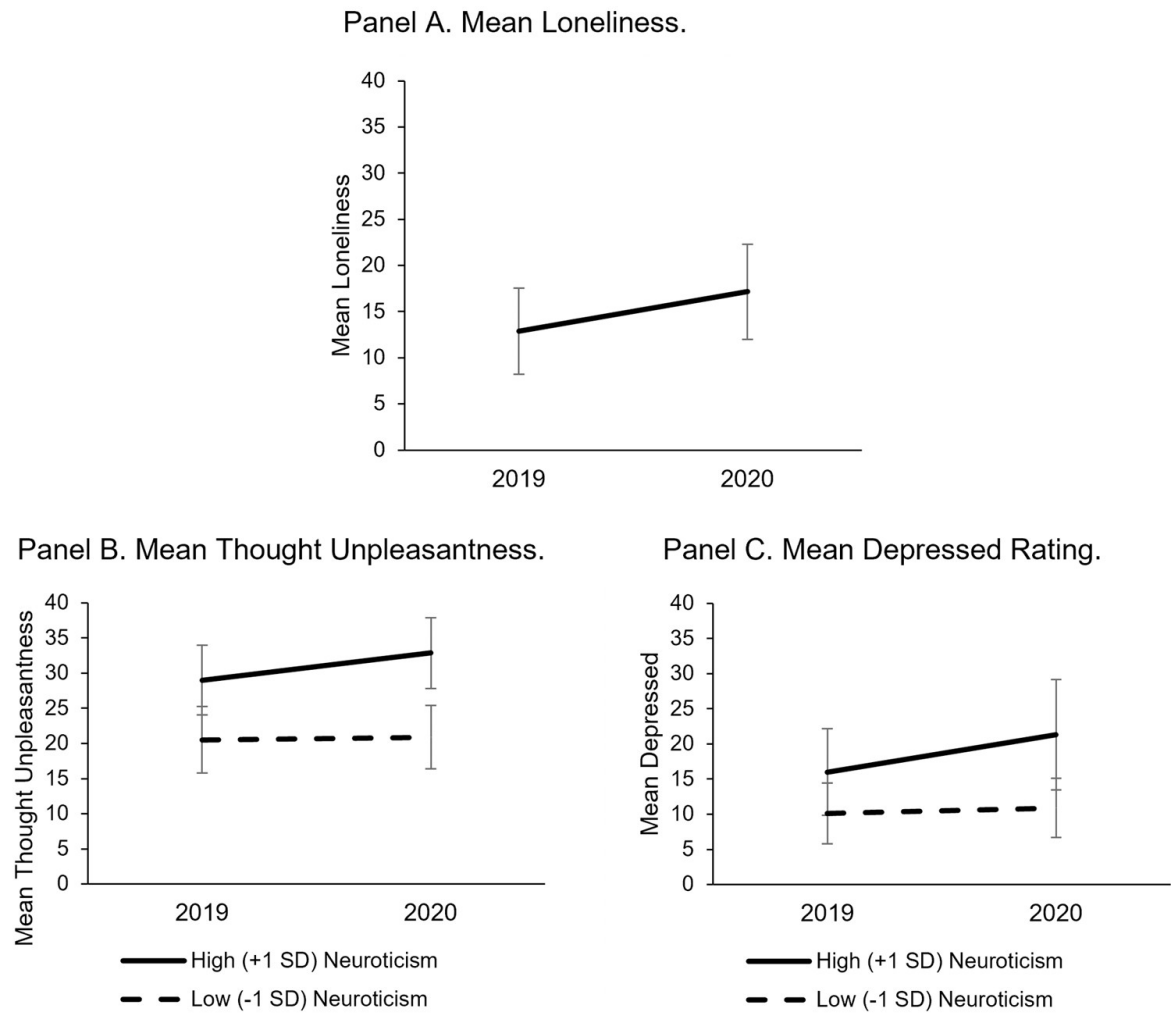
Mean level changes in loneliness, thought unpleasantness, and depressed. Mean scores on ecological momentary assessment measures are presented for 2019 and 2020 with 95% confidence intervals. In panels B and C, high neuroticism was defined as neuroticism scores one standard deviation above the sample mean and low neuroticism was defined as neuroticism scores one standard deviation below the sample mean.

S1 Table in the Supplemental Material shows full model results. Thought unpleasantness, stress, mood valence, thought control, worry, tense/anxious, depressed and frustrated did not show significant change, but the average momentary loneliness (*B* = 4.28, 95% Confidence Interval [1.23, 7.34], *p* = .006) significantly increased from 2019 to 2020. Trait neuroticism moderated within-person changes in thought unpleasantness and depressive feelings. Specifically, those higher in neuroticism increased more in thought unpleasantness (*B* = 2.56, 95% CI [0.18, 4.93], *p* = .03) and feeling depressed (*B* = 3.36, 95% CI [0.28, 6.44], *p* = .03) from 2019 to 2020 relative to those lower in trait neuroticism (see Fig 1). MCI did not significantly moderate within-person change for any outcome.

Effect sizes for significant results were calculated using pseudo R^2^ to determine the proportion of variance explained in the outcome variable. The COVID onset variable explained 2.88% of the level 1 variance in the loneliness variable. For the significant interaction effects, neuroticism explained 4.33% of the variance in the COVID onset slope for the thought unpleasantness outcome and 1.12% of the variance in the COVID onset slope for the depressed outcome.

A priori power analyses were not conducted because when the longitudinal study began in 2017, it was not designed to answer questions about an unexpected pandemic. To provide the clearest within-person comparison and limit the influence of other major societal events later in 2020, the sample size in the current analyses was limited to participants that completed burst periods both before and during the early pandemic. To provide additional insight regarding the observed significant effects, we applied the multilevel models reported above to 1,000 randomly sampled datasets drawn from the full sample size in the observed dataset. The multilevel model with loneliness as the outcome variable was conducted on each sampled dataset, each of which included 10 randomly selected participants from the observed data. The within-person change in mean loneliness rating from 2019 to 2020 (i.e., the fixed effect of COVID onset) was significant in 87.2% of the 1,000 simulated datasets with a sample size of 10 participants. This suggests a relatively robust effect observed in the full dataset for within-person change in loneliness. This process was repeated for the models that showed significant COVID onset X neuroticism effects. For the thought unpleasantness outcome, the COVID onset X neuroticism effect was significant in 66.4% of the 1,000 simulated datasets with a sample size of 10 participants. For the feeling depressed outcome, the COVID onset X neuroticism effect was significant in 66.5% of the 1,000 simulated datasets with a sample size of 10 participants.

### Longitudinal community-level psychological well-being

**Fig 2:**
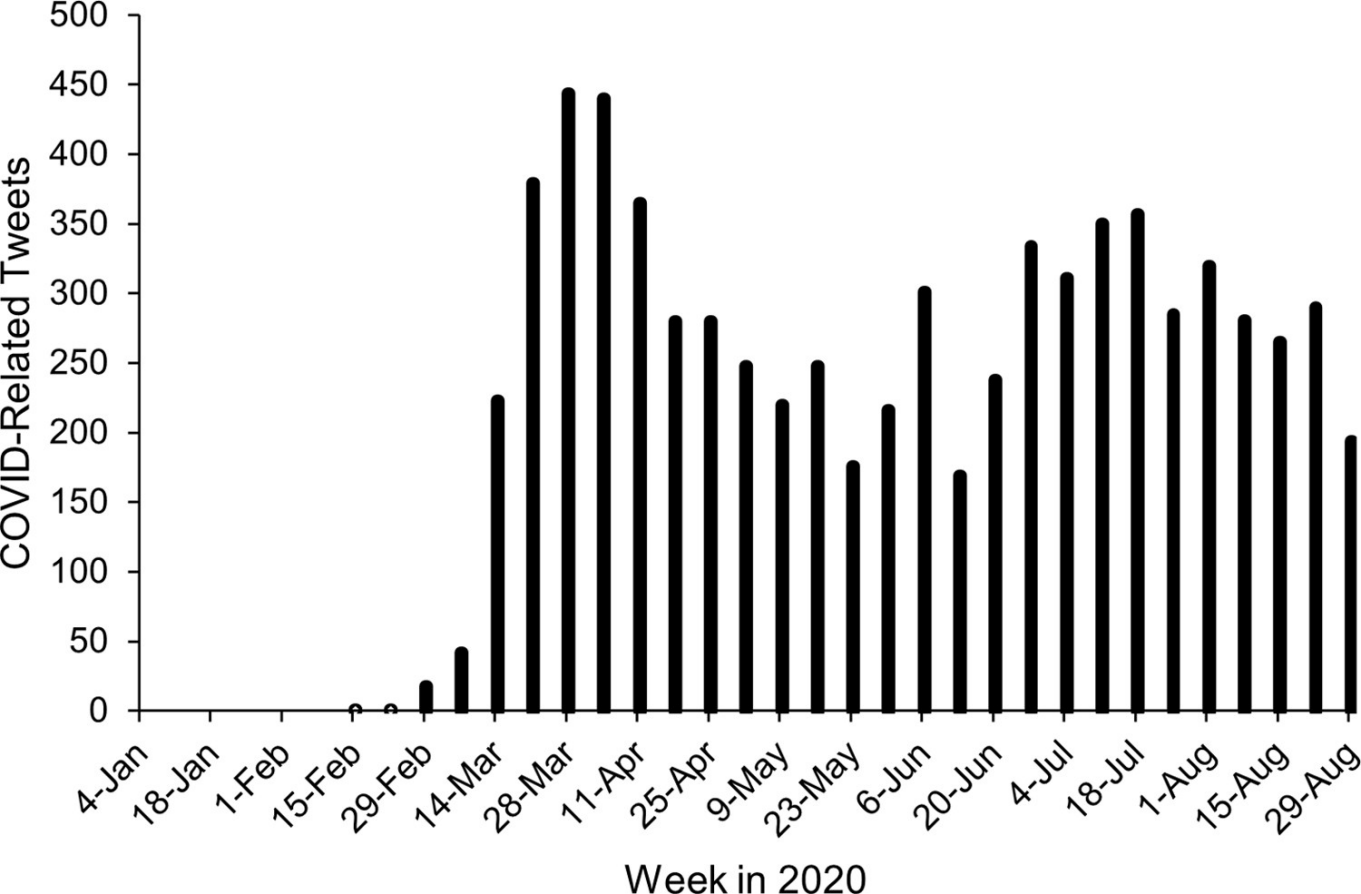
COVID-related tweets in Bronx County in 2019 and 2020. The dates along the x-axis represent the midpoint of the week in 2020. Counts for weeks in 2019 are not depicted because no COVID-related tweets were detected.

Fig 2 shows COVID-related tweet counts for each week between January 1 and September 1 in 2020. These weeks were selected to include the 19 weeks of 2020 when individual-level EMA data were collected (February 5-June 16). As seen in Fig 2, COVID-related tweets appeared in mid-February and rapidly increased across March.

**Fig 3:**
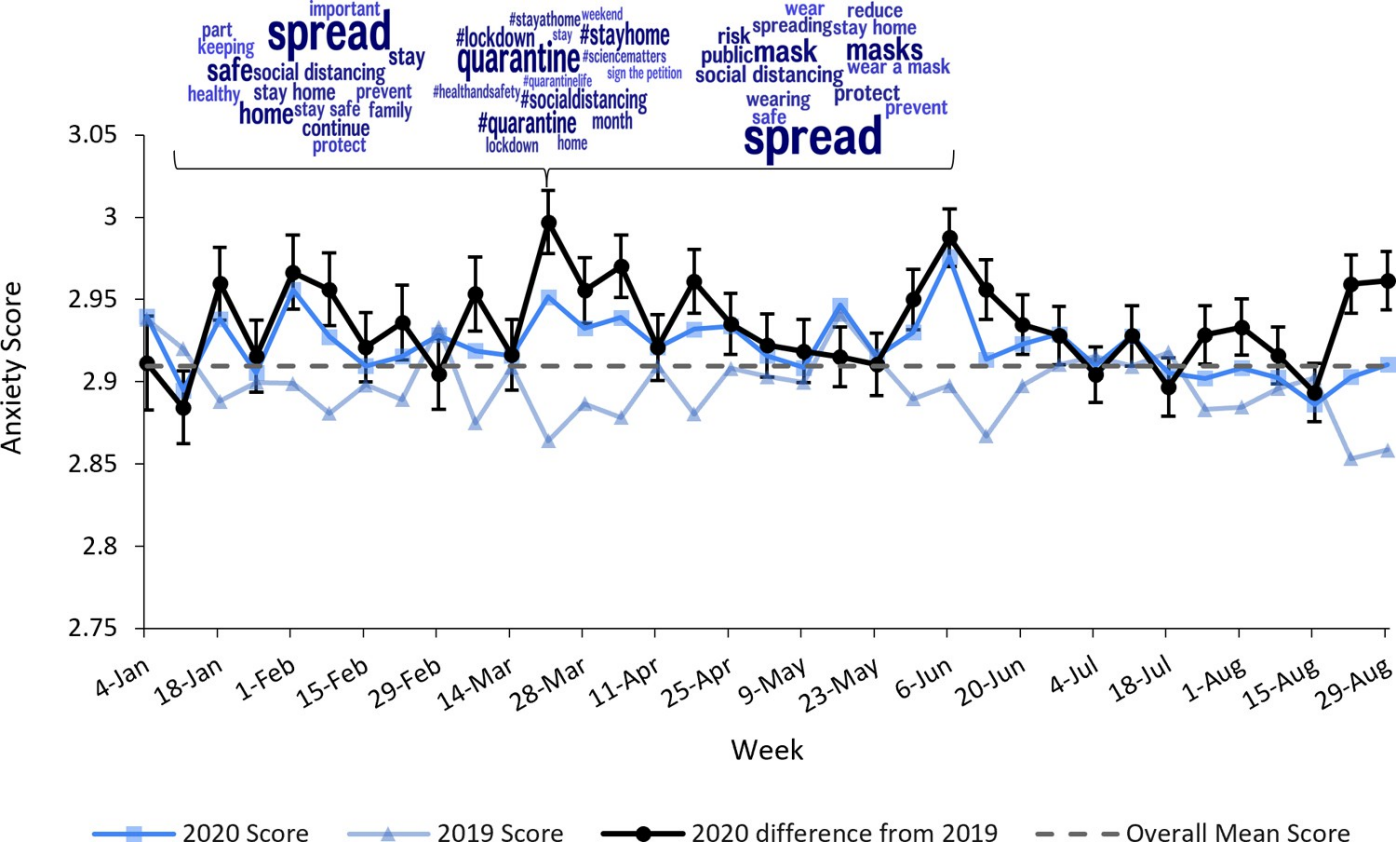
Weekly linguistic anxiety score in 2019 and 2020. Comparisons of Bronx County community anxiety scores between corresponding weeks in 2019 and 2020 are shown. The points along each line represent weeks 0–34 in 2019 and 2020 and the dates along the x-axis represent the midpoint of the week in 2020. The black line with circle markers at each week represents the difference between 2020 and 2019 scores (centered on the overall mean score) and the 95% confidence intervals are included. Word clouds derived from COVID-related Bronx County Tweets are shown for week 11 ranging from March 18, 2020 to March 24, 2020.

**Fig 4:**
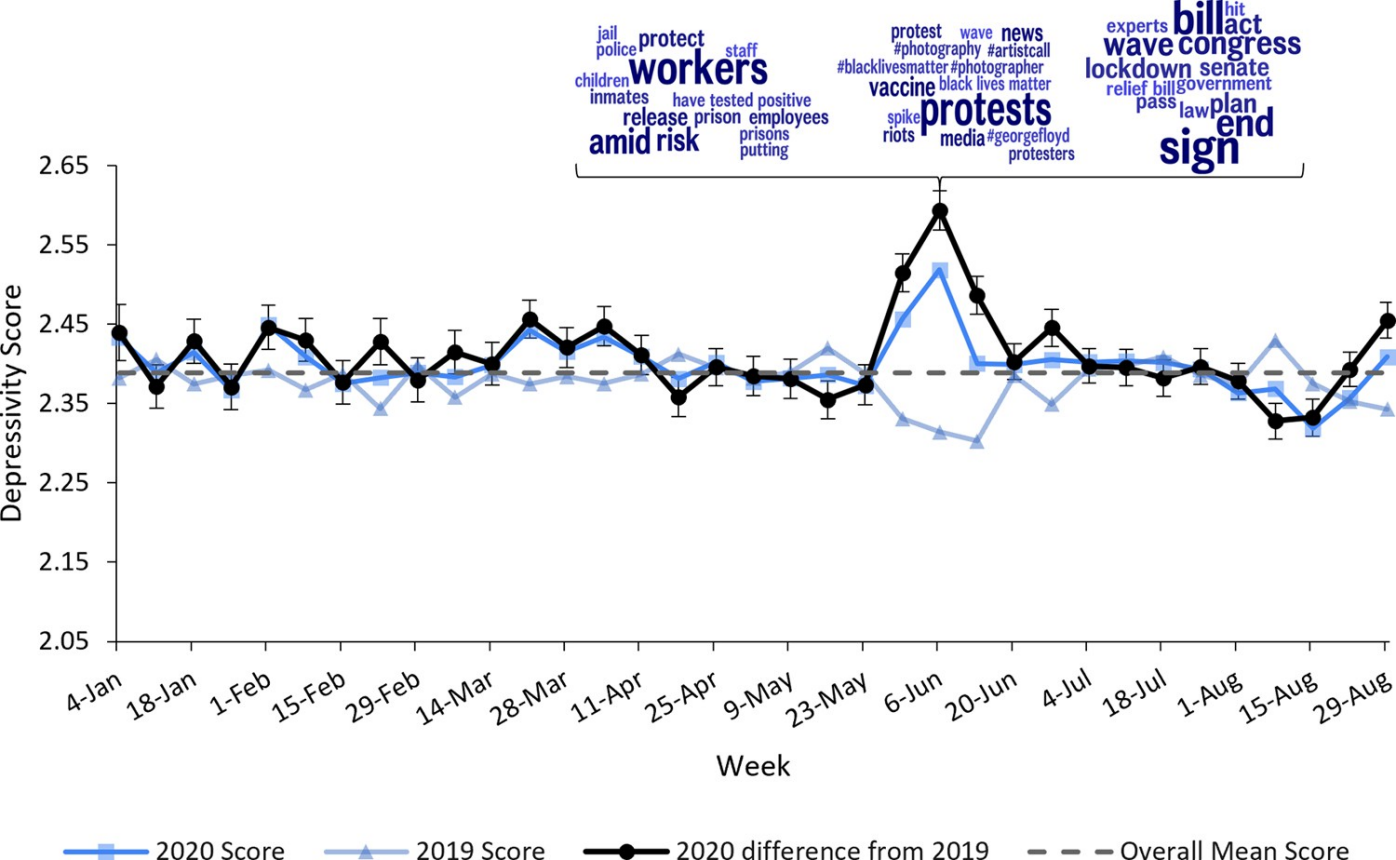
Weekly linguistic depressivity in 2019 and 2020. Comparisons of Bronx County community depressivity scores between corresponding weeks in 2019 and 2020 are shown. The points along each line represent weeks 0–34 in 2019 and 2020 and the dates along the x-axis represent the midpoint of the week in 2020. The black line with circle markers at each week represents the difference between 2020 and 2019 scores (centered on the overall mean score) and the 95% confidence intervals are included. Word clouds derived from COVID-related Bronx County Tweets are shown for week 22 ranging from June 3, 2020 to June 9, 2020.

**Fig 5:**
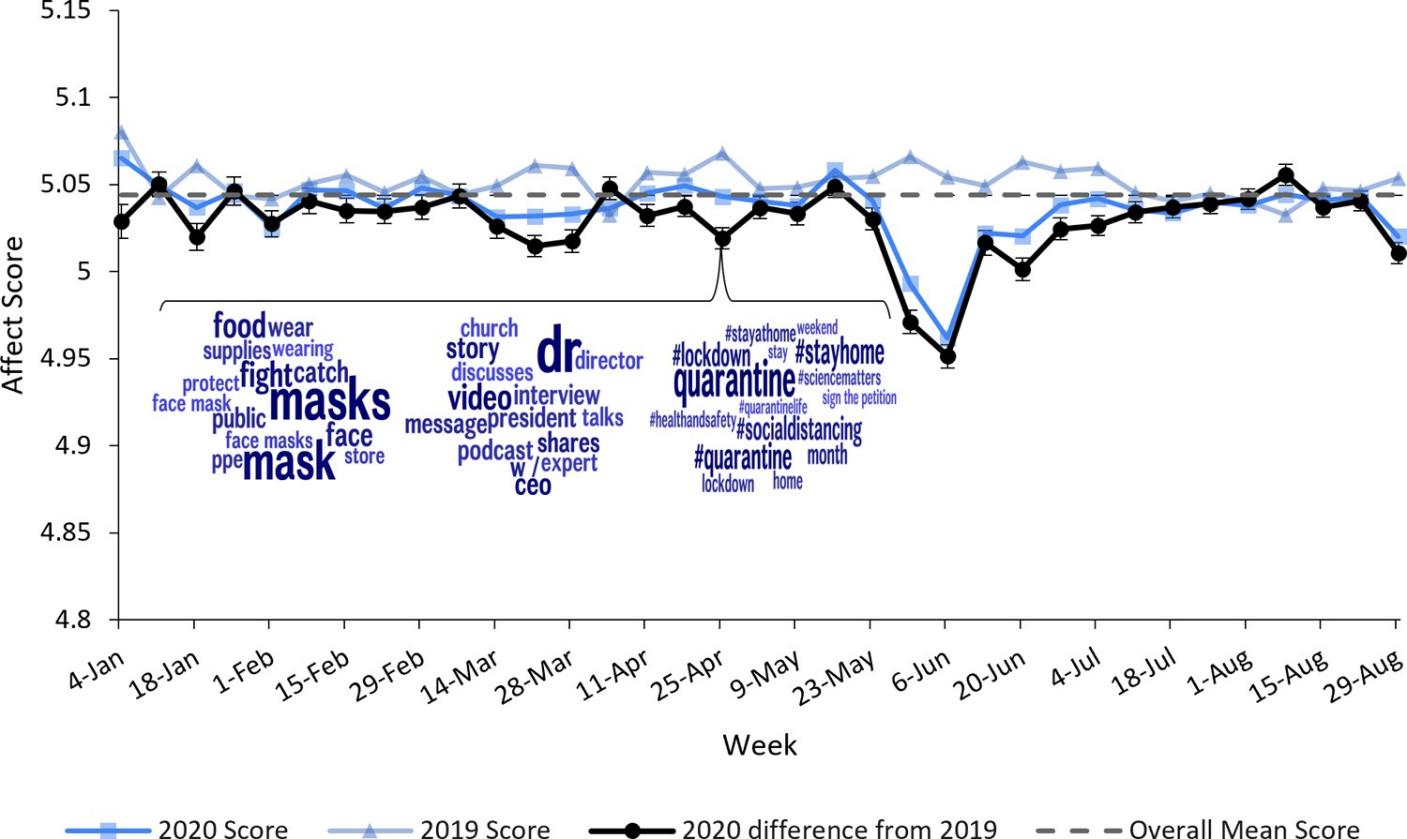
Weekly linguistic affect in 2019 and 2020. Comparisons of Bronx County community affect scores between corresponding weeks in 2019 and 2020 are shown. The points along each line represent weeks 0–34 in 2019 and 2020 and the dates along the x-axis represent the midpoint of the week in 2020. The black line with circle markers at each week represents the difference between 2020 and 2019 scores (centered on the overall mean score) and the 95% confidence intervals are included. Word clouds derived from COVID-related Bronx County Tweets are shown for week 16 ranging from April 22, 2020 to April 28, 2020.

Figs 3–5 depict continuous community markers for Bronx County extracted from Twitter between January 1 and September 1 in 2019 and 2020. Fig 3 shows community anxiety scores, Fig 4 shows community depressivity scores, and Fig 5 shows community affect scores in Bronx County. In Figs 3–5, each point represents the average score for each week. The dashed line shows the mean score across all weeks in both years. The circle markers (black bold line) show the difference between 2020 and 2019 for each week, centered on the mean score (dashed line) with the 95% CI included. Any weeks in which the black circle markers are above this dashed line indicate community scores which were higher in 2020 relative to 2019, and those weeks below the dashed line indicate community scores which were lower in 2020 than 2019. Community anxiety scores and the 95% CI were higher in 2020 relative to 2019 for 11 of 19 EMA-aligned weeks (58%) examined (see Fig 3), community depressivity scores and the 95% CI were higher for 8 of 19 EMA-aligned weeks (42%) in 2020 (Fig 4), and community affect scores and the 95% CI were more negatively-valenced for 14 of 19 EMA-aligned weeks (74%) in 2020 (Fig 5). COVID-19 topic word clouds were overlaid on these panels so that the frequently used words or phrases in Tweets could be explored to illuminate events or aspects of the pandemic period that may have contributed to increases in community anxiety, depressivity, or affect valence.

### Comparing individual-level and community-level data

**Fig 6:**
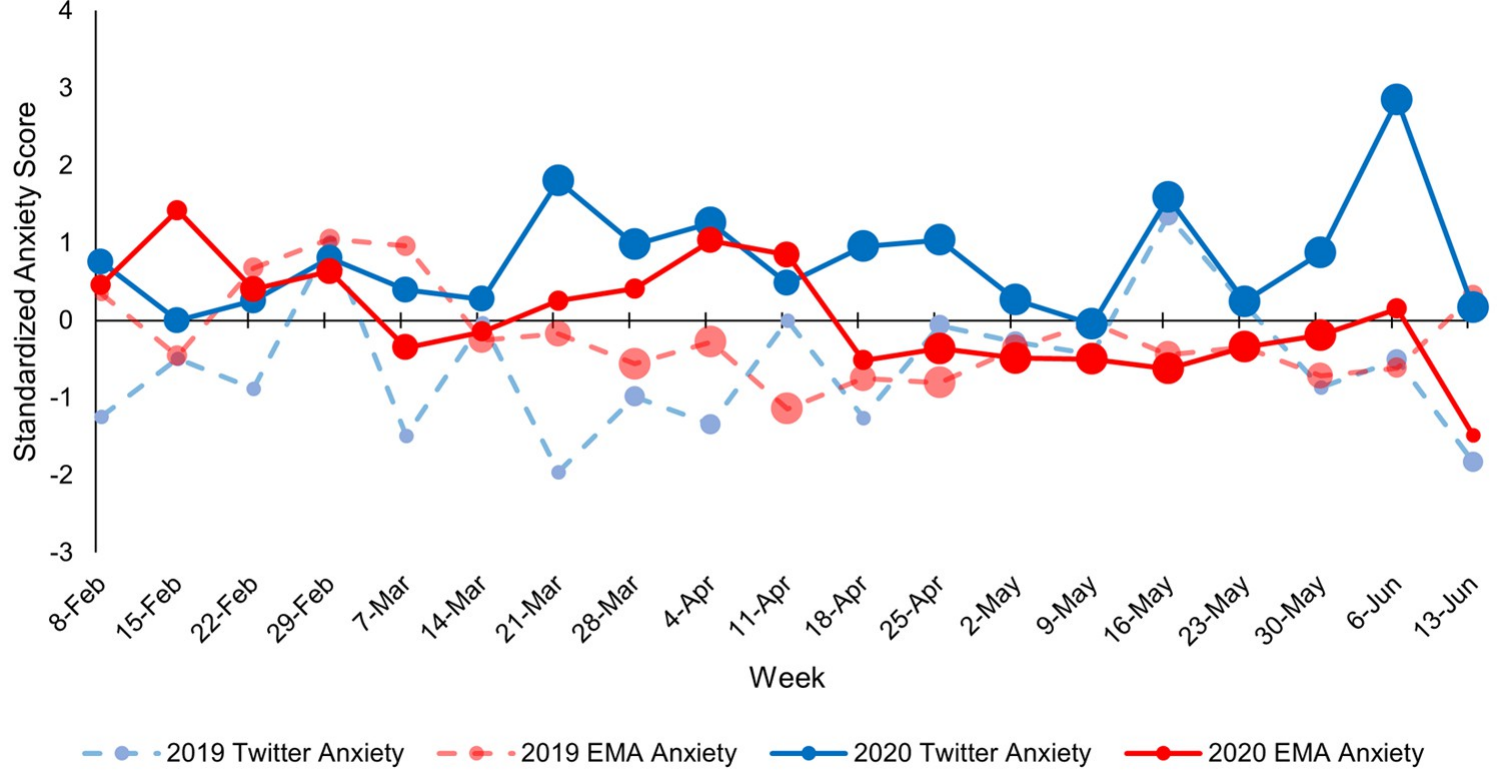
Comparison of standardized individual-level and community-level anxiety scores. Weekly scores were standardized across all weeks for which both individual-level and community-level data were available (32 weeks in 2019 and 19 weeks in 2020). The dates on the x-axis are the midpoint of each week. Individual-level weekly scores are presented in red, and community-level weekly scores are presented in blue. Scores from 2019 are on the dashed and faded lines whereas scores from 2020 are on the solid lines. The size of the points on each line corresponds to the number of EAS participants (for the red lines) and the number of Twitter users (for the blue lines) at each week divided into quartiles with bigger points representing more individuals. The number of participants contributing EMA data during each week ranged from 1–35 and the number of Twitter users at each week ranged from 673–1,869. S1 Fig in the Supplemental Material shows the number of EAS participants and Twitter users for each week in 2019 and 2020.

**Fig 7:**
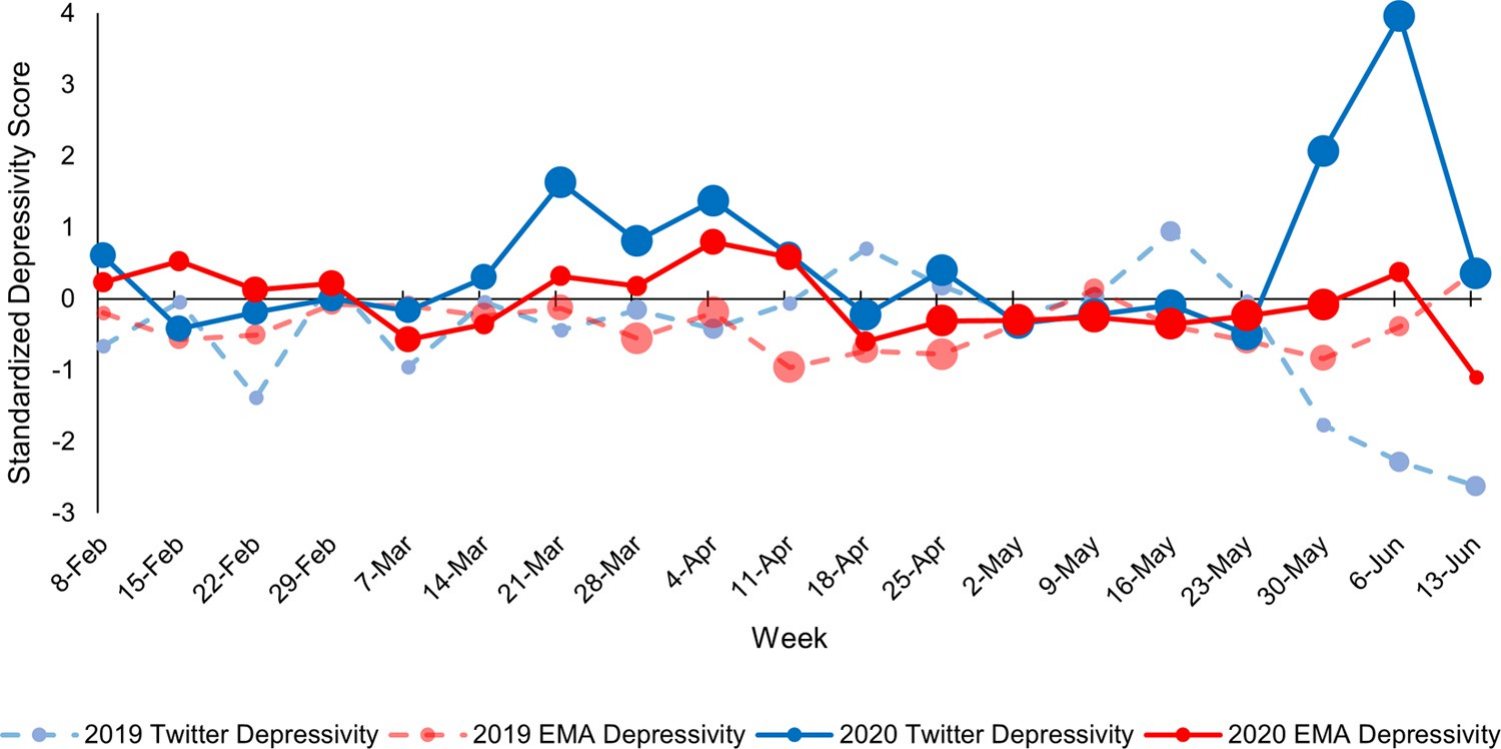
Comparison of standardized individual-level and community-level depressivity scores. Weekly scores were standardized across all weeks for which both individual-level and community-level data were available (32 weeks in 2019 and 19 weeks in 2020). The dates on the x-axis are the midpoint of each week. Individual-level weekly scores are presented in red, and community-level weekly scores are presented in blue. Scores from 2019 are on the dashed and faded lines whereas scores from 2020 are on the solid lines. The size of the points on each line corresponds to the number of EAS participants (for the red lines) and the number of Twitter users (for the blue lines) at each week divided into quartiles with bigger points representing more individuals. The number of participants contributing EMA data during each week ranged from 1–35 and the number of Twitter users at each week ranged from 673–1,869. S1 Fig in the Supplemental Material shows the number of EAS participants and Twitter users for each week in 2019 and 2020.

**Fig 8:**
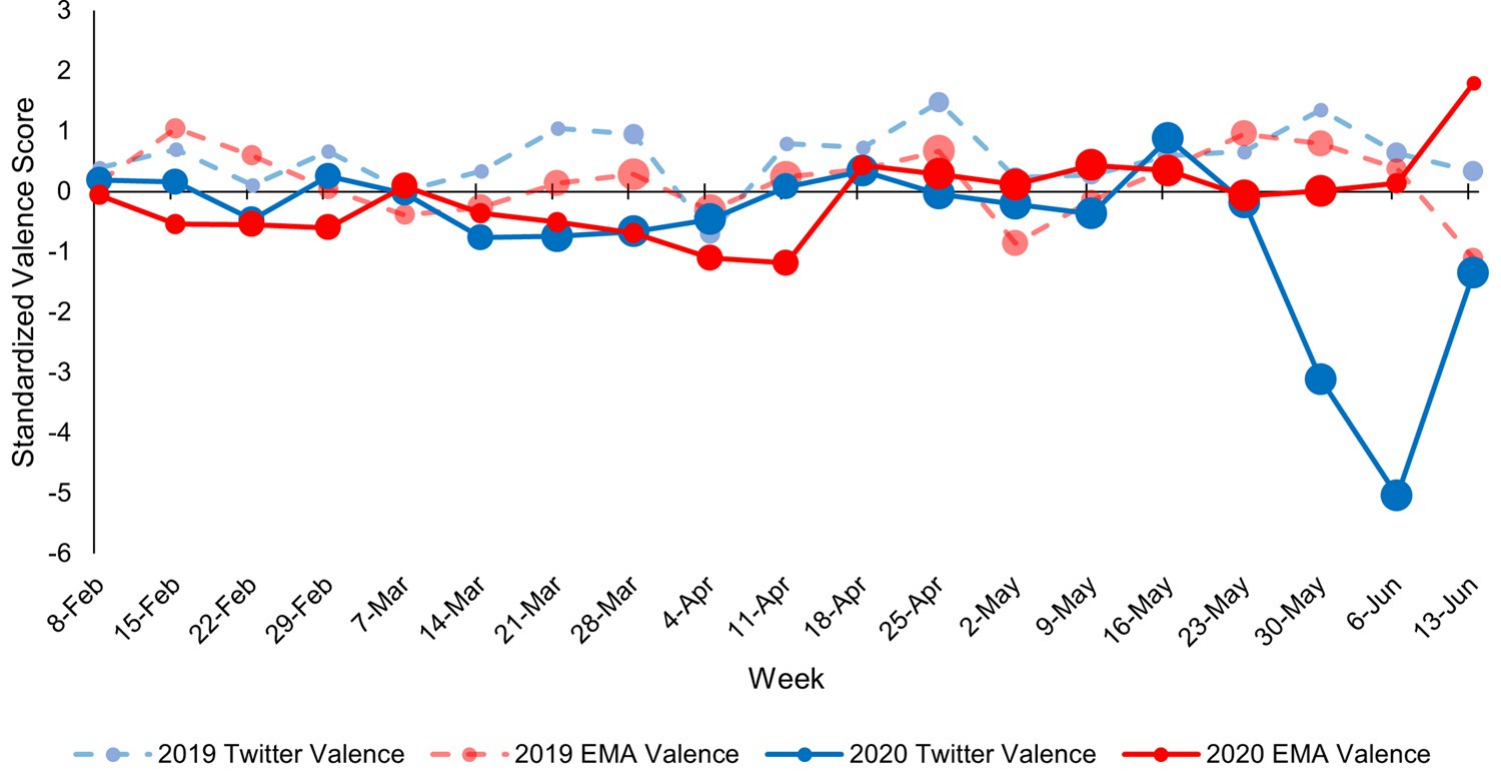
Comparison of standardized individual-level and community-level valence scores. Weekly scores were standardized across all weeks for which both individual-level and community-level data were available (32 weeks in 2019 and 19 weeks in 2020). The dates on the x-axis are the midpoint of each week. Individual-level weekly scores are presented in red, and community-level weekly scores are presented in blue. Scores from 2019 are on the dashed and faded lines whereas scores from 2020 are on the solid lines. The size of the points on each line corresponds to the number of EAS participants (for the red lines) and the number of Twitter users (for the blue lines) at each week divided into quartiles with bigger points representing more individuals. The number of participants contributing EMA data during each week ranged from 1–35 and the number of Twitter users at each week ranged from 673–1,869. S1 Fig in the Supplemental Material shows the number of EAS participants and Twitter users for each week in 2019 and 2020.

Individual-level EMA data were aggregated for each week in 2019 and 2020 for which data were available and aligned with community-level data. The correlation between the individual-level data on three outcomes (tense/anxious, depressed, and mood valence) and the corresponding community-level data on each of these outcomes (linguistic anxiety, linguistic depressivity, and linguistic affect) were calculated for 2019 and 2020. Figs 6–8 show the standardized individual-level and community-level weekly scores for 2019 and 2020. Fig 6 shows weekly anxiety scores, Fig 7 shows weekly depressivity scores, and Fig 8 shows weekly affect and mood valence scores. The correlation between individual-level and community-level weekly scores were calculated for the weeks in which data from both sources aligned–32 weeks in 2019 and 19 weeks in 2020. The correlation between weekly scores of anxiety was *r* = -.19, *p* = .31 in 2019 and *r* = .09, *p* = .70 in 2020. The correlation between weekly scores of depressivity was *r* = -.24, *p* = .19 in 2019 and *r* = .39, *p* = .10 in 2020. The correlation between weekly scores of affect and mood valences was *r* = .21, *p* = .25 in 2019 and *r* = -.16, *p* = .52 in 2020.

## Discussion

This study presents a novel approach of contextualizing longitudinal changes in psychological well-being during the early COVID-19 pandemic by integrating individual-level EMA data with community-level indicators. At the individual-level, we found within-person increases in loneliness from 2019 to 2020 among EAS older adult participants. We also found that participants higher (*vs*. lower) in neuroticism showed more within-person increases from 2019 to 2020 in momentary thought unpleasantness and feeling depressed. At the community-level, language analysis of Tweets from Bronx County where EAS participants resided showed declines in well-being. Specifically, higher anxiety and depressivity and more negatively valenced affect were observed in community Tweets in the majority of 2020 weeks relative to the same 2019 weeks. Finally, the topics revealed in word clouds derived from Twitter were also used to elucidate the week-to-week fluctuations observed in both the individual-level and the surrounding community across 2019 and 2020. These topics both support the interpretation of pandemic-related threats to well-being at both individual and community levels, as well as other major disruptions that occurred during 2020.

It is important to consider that although the early pandemic period is likely a new exemplar for the classic developmental definitions of a shared history-graded influence [45], it did not impact all individuals in the same way. Indeed, multilevel models of individual-level data fit better when individual differences in the patterns of change from 2019 to 2020 were allowed and neuroticism emerged as a significant moderator of change. Considering the goal of identifying generalizable patterns in research, this study’s results suggest caution in this pursuit—patterns of the pandemic’s impact on psychological well-being are expected to differ across communities (and samples), unfolding at different times and with different triggers driven by local conditions. The community-level data showed varying levels of anxiety, depressivity, and negative affect (relative to average levels) across weeks and the word clouds representing common topics posted on Twitter provide insight into the different events that may have caused the fluctuating levels of community-level psychological well-being. Some of the largest differences between 2020 and 2019 observed in the community-level markers of depressivity and negatively-valenced affect occurred in early June 2020. Selected word clouds for this week highlight new topics (e.g., racism, protests, #blacklivesmatter), which did not appear as common topics earlier in 2020 and corresponded to major non-COVID events such as the murder of George Floyd and the incident in which a white woman called the police on a Black man birdwatching [46,47]. Sampling from a single week or aggregating across communities and weeks would likely have missed this important nuance. The use of Twitter demonstrated an approach for using auxiliary data to improve understanding of the social environment within a geographic area in psychological survey research without increasing burden on participants.

Despite the lack of significant correlations between weekly scores, results shown in Figs 6–8 highlight the week-to-week variability and different patterns across psychological well-being measures between EAS participants and their surrounding community environment. For example, community and individual depressivity scores during the weeks of March and early-April 2020 appear to be tightly coupled and covary across these weeks (see Fig 7) which corresponds to the period when COVID-19-related cases, hospitalizations, and deaths peaked in New York City [48]. The variability across weeks in 2020 among both individual-level and community-level scores highlight the heterogeneity of the early pandemic period. Future studies that analyze data collected during 2020, but do not attend to date or location of data collection, may be influenced by an increasing mix of other significant and stressful events in an especially eventful year (e.g., changes in COVID-19 rates and related restrictions, protests, presidential election), with some locations and communities likely to be affected more by these events as well. For example, because the EAS sample includes only older adults, participants’ psychological well-being may not have been as tightly tied to community changes in services impacting age-relevant roles, such as school closures and childcare which may have been picked up in Twitter [1,49]. Considering the local conditions during the early pandemic period (e.g., COVID-19 case count, stay-at-home orders) provides relevant context for understanding data collected on individuals. With few exceptions [50,51], most psychological studies have not considered the local conditions to contextualize individual-level data.

Individual-level data were limited to individuals over age 70 residing in a single county and community-level data were drawn from Twitter users from the same county, during the same time periods. Although these narrowly focused datasets and time periods may limit our findings’ generalizability, these choices were made to enhance validity by cleanly aligning individuals with their community in real-time. As mentioned above, demographics and community context likely influenced how individuals experienced the early pandemic, and thus it is valuable to examine targeted groups whose experiences may not match those that would be concluded from studies that group an entire population together as a whole. In illustration of this concept, the age of our participants may help explain why more widespread changes in psychological well-being from 2019 to 2020 did not emerge in our study, considering previous findings that older adults have experienced less severe impairments of their psychological well-being due to COVID [10,11]. Even with our targeted time scope, though, we dichotomized time into “pre-pandemic” (2019) and “pandemic” (2020) periods, thus treating the pandemic period as uniform and not further differentiating how the impacts of COVID-19 may have changed within this period. In future work, we will use the Twitter-based indicators as time-varying predictors, allowing us to model the effects of changing community distress on individual outcomes. Future research assessing the disruptive effects of the pandemic and other history-graded influences should consider how these effects differ across time and space and how they interact with other concurrent societal events.

## Conclusions

Research on the impact of the COVID-19 pandemic on psychological well-being should consider the time and place of data collection. This study showed that from February to June of 2020, community levels of anxiety, depressivity, and negative affect fluctuated from week to week, suggesting that this period should not be considered homogenous. Further analysis of longitudinal reports of momentary psychological well-being collected from older adults living in Bronx County before and during the pandemic suggest increases in loneliness and poorer general psychological well-being specifically among individuals higher in neuroticism during the pandemic. Future work should consider critical community-level variables around current events such as case count, policies to prevent the spread of cases, and available information regarding risk in addition to community emotion levels. Additionally, the impact of social events such as protests of police killings and the U.S. presidential election that occurred during 2020 should also be considered. Together, the findings from individual-level and community-level data present an in-depth assessment of older adults’ psychological well-being within a specific community during the early months of the pandemic, while being mindful of individuals’ temporal, spatial, and social contexts.

## Supporting information

S1 Fig Weekly Twitter users and weekly participants in the Einstein Aging Study (EAS) included in the study period. *Note*. EAS = Einstein Aging Study. Counts of Twitter users and EAS participants are presented for 2019 and 2020. The data were aligned at the respective weeks for 2019 and 2020 which do not correspond to the exact same dates in each year. The dates displayed on the x-axis represent the midpoint of the week in 2020. In Panel B, individual EAS participants completed EMA surveys across multiple weeks and therefore are included in the bars across multiple weeks meaning the sum of these bars exceeds the number of EAS participants (N = 78). Two participants’ data are not depicted in this graph because one participant completed their pre-COVID EMA burst in December 2018, another in October 2019. These participants’ observations, however, were included in the analyses of individual-level data. In the EMA portion of the results for this manuscript, we limited our 2020 time window to data completed during the week of June 13, 2020. Thus, the orange bars in panel B do not continue past the week of June 13. The x-axis extends beyond the 2020 data used in our analyses to show the EMA 2019 data collection, as well as to mirror the x-axis in the Twitter data.
(TIF)

S1 Table Full multilevel model results. Results of separate parallel two-level multilevel models are shown. A total of 7,329 observations from 78 participants were collected in 2019 and a total of 7,204 observations from 78 participants were collected in 2020. One participant did not complete the personality measures and thus these 197 observations were excluded from these models. The number of observations used in each model differs because each outcome was assessed at different frequencies. Thought unpleasantness and stress were assessed 6 times per day and thought control, worry, tense/anxious, lonely, depressed, and frustrated were assessed 5 times per day.
(DOCX)
