# Supplementary figures and images for: The where and when of COVID-19: Using ecological and Twitter-based assessments to examine impacts in a temporal and community context
Source: PLoS One. 2022 Feb 23;17(2):e0264280. doi: 10.1371/journal.pone.0264280 (PMC8865674; doi:10.1371/journal.pone.0264280)

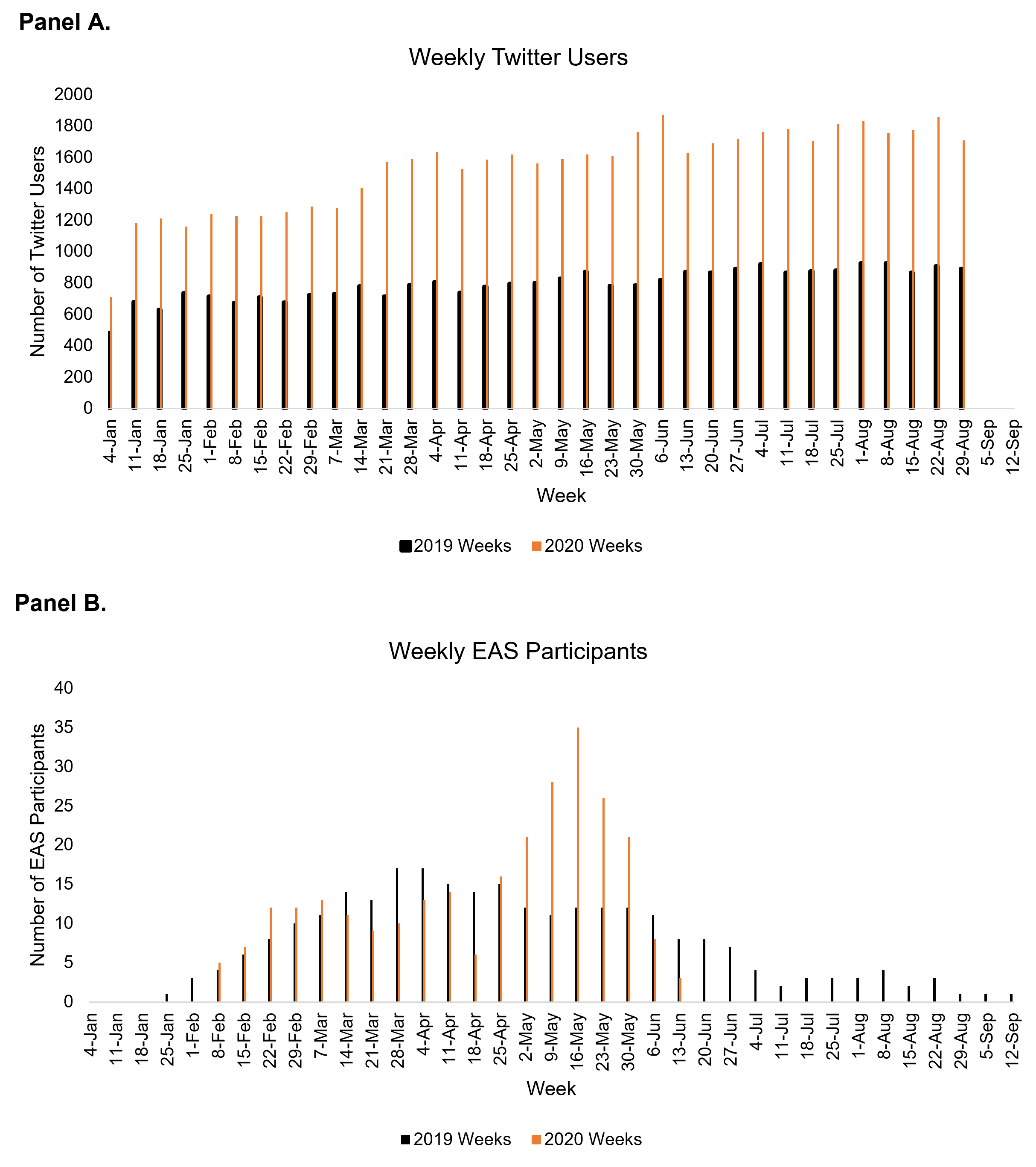

Supplement: S1 Fig — Note. EAS = Einstein Aging Study. Counts of Twitter users and EAS participants are presented for 2019 and 2020. The data were aligned at the respective weeks for 2019 and 2020 which do not correspond to the exact same dates in each year. The dates displayed on the x-axis represent the midpoint of the week in 2020. In Panel B, individual EAS participants completed EMA surveys across multiple weeks and therefore are included in the bars across multiple weeks meaning the sum of these bars exceeds the number of EAS participants (N = 78). Two participants’ data are not depicted in this graph because one participant completed their pre-COVID EMA burst in December 2018, another in October 2019. These participants’ observations, however, were included in the analyses of individual-level data. In the EMA portion of the results for this manuscript, we limited our 2020 time window to data completed during the week of June 13, 2020. Thus, the orange bars in panel B do not continue past the week of June 13. The x-axis extends beyond the 2020 data used in our analyses to show the EMA 2019 data collection, as well as to mirror the x-axis in the Twitter data. (TIF) [file pone.0264280.s001.tif]
