# Supplementary material for: The where and when of COVID-19: Using ecological and Twitter-based assessments to examine impacts in a temporal and community context
Source: PLoS One. 2022 Feb 23;17(2):e0264280. doi: 10.1371/journal.pone.0264280 (PMC8865674; doi:10.1371/journal.pone.0264280)
Supplement: S1 Table — Results of separate parallel two-level multilevel models are shown. A total of 7,329 observations from 78 participants were collected in 2019 and a total of 7,204 observations from 78 participants were collected in 2020. One participant did not complete the personality measures and thus these 197 observations were excluded from these models. The number of observations used in each model differs because each outcome was assessed at different frequencies. Thought unpleasantness and stress were assessed 6 times per day and thought control, worry, tense/anxious, lonely, depressed, and frustrated were assessed 5 times per day. (DOCX) [file pone.0264280.s002.docx]

**Table S1.** Multilevel model results.

|  | Negative Thoughts | | | | | | | | | Subjective Stress | | |
| --- | --- | --- | --- | --- | --- | --- | --- | --- | --- | --- | --- | --- |
|  | Thought Unpleasantness | | | Thought Control | | | Worry | | | Stress | | |
|  | B | SE | *p* | B | SE | *p* | B | SE | *p* | B | SE | *p* |
| **Models with Covariates** |  |  |  |  |  |  |  |  |  |  |  |  |
| *Fixed Effects* |  |  |  |  |  |  |  |  |  |  |  |  |
| Intercept | 25.28 | 1.96 | <.001 | 16.60 | 2.27 | <.001 | 23.18 | 2.35 | <.001 | 20.00 | 2.13 | <.001 |
| COVID Onset | 1.10 | 1.03 | 0.28 | 2.33 | 1.33 | 0.08 | 0.22 | 1.37 | 0.87 | 0.50 | 1.16 | 0.67 |
| Gender | 1.46 | 2.81 | 0.61 | -2.08 | 3.14 | 0.51 | -1.59 | 3.57 | 0.66 | -0.02 | 3.36 | 0.99 |
| Age | -0.24 | 0.24 | 0.32 | -0.33 | 0.27 | 0.23 | -0.16 | 0.33 | 0.63 | -0.16 | 0.26 | 0.53 |
| MCI | -4.63 | 3.66 | 0.21 | 3.20 | 3.85 | 0.41 | 0.26 | 4.10 | 0.95 | -2.04 | 4.07 | 0.62 |
| Extraversion | -3.28 | 1.97 | 0.10 | -0.80 | 2.57 | 0.76 | -3.51 | 2.80 | 0.21 | -0.64 | 2.34 | 0.78 |
| Neuroticism | 7.55 | 2.10 | <.001 | 3.19 | 2.34 | 0.18 | 5.71 | 2.56 | 0.03 | 7.42 | 2.62 | 0.01 |
| *Random Effects* |  |  |  |  |  |  |  |  |  |  |  |  |
| Intercept | 174.60 | 29.83 | <.001 | 204.79 | 34.82 | <.001 | 250.33 | 42.65 | <.001 | 224.32 | 38.06 | <.001 |
| COVID Onset | 77.74 | 13.35 | <.001 | 132.99 | 22.58 | <.001 | 137.51 | 23.61 | <.001 | 98.16 | 16.80 | <.001 |
| Residual | 183.96 | 2.18 | <.001 | 190.08 | 2.49 | <.001 | 249.68 | 3.28 | <.001 | 230.55 | 2.74 | <.001 |
| **Models with Personality Interactions** |  |  |  |  |  |  |  |  |  |  |  |  |
| *Fixed Effects* |  |  |  |  |  |  |  |  |  |  |  |  |
| Intercept | 24.77 | 2.02 | <.001 | 16.61 | 2.27 | <.001 | 23.21 | 2.35 | <.001 | 19.89 | 2.16 | <.001 |
| COVID Onset | 2.12 | 1.26 | 0.09 | 2.56 | 1.56 | 0.10 | -0.15 | 1.65 | 0.93 | 0.87 | 1.54 | 0.57 |
| Gender | 1.46 | 2.81 | 0.60 | -2.08 | 3.14 | 0.51 | -1.60 | 3.57 | 0.66 | -0.01 | 3.36 | 0.99 |
| Age | -0.24 | 0.24 | 0.32 | -0.32 | 0.27 | 0.23 | -0.16 | 0.33 | 0.63 | -0.16 | 0.26 | 0.53 |
| MCI | -2.77 | 3.73 | 0.46 | 3.20 | 3.86 | 0.41 | 0.15 | 4.07 | 0.97 | -1.62 | 4.06 | 0.69 |
| COVID Onset X MCI | -3.66 | 1.96 | 0.06 | -0.83 | 3.35 | 0.80 | 1.30 | 3.26 | 0.69 | -1.35 | 2.16 | 0.53 |
| Extraversion | -2.15 | 2.23 | 0.34 | -0.82 | 2.57 | 0.75 | -3.70 | 2.81 | 0.19 | -0.63 | 2.40 | 0.79 |
| COVID Onset X Extraversion | -2.25 | 1.53 | 0.14 | -0.50 | 1.42 | 0.73 | 2.28 | 1.66 | 0.17 | -0.05 | 1.77 | 0.98 |
| Neuroticism | 6.27 | 2.13 | <.01 | 3.28 | 2.34 | 0.17 | 5.56 | 2.53 | 0.03 | 6.66 | 2.61 | 0.01 |
| COVID Onset X Neuroticism | 2.56 | 1.21 | 0.03 | 2.24 | 2.24 | 0.20 | 1.94 | 2.11 | 0.36 | 2.59 | 1.67 | 0.12 |
| *Random Effects* |  |  |  |  |  |  |  |  |  |  |  |  |
| Intercept | 173.36 | 29.45 | <.001 | 204.82 | 34.82 | <.001 | 250.42 | 42.67 | <.001 | 224.50 | 38.11 | <.001 |
| COVID Onset | 72.55 | 12.78 | <.001 | 134.34 | 23.26 | <.001 | 140.18 | 24.56 | <.001 | 98.95 | 17.27 | <.001 |
| Residual | 183.96 | 2.18 | <.001 | 190.08 | 2.49 | <.001 | 249.68 | 3.28 | <.001 | 230.55 | 2.74 | <.001 |

**Table S1 (Continued)**. Multilevel Model Results.

|  |  | Negative Affect | | | | | | | | | | | |  |  |  | |
| --- | --- | --- | --- | --- | --- | --- | --- | --- | --- | --- | --- | --- | --- | --- | --- | --- | --- |
|  |  | Tense/Anxious | | | Lonely | | | Depressed | | | Frustrated | | | Mood Valence | | | |
|  |  | B | SE | *p* | B | SE | *p* | B | SE | *p* | B | SE | *p* | B | SE | *p* |  |
| **Models with Covariates** |  |  |  |  |  |  |  |  |  |  |  |  |  |  |  |  |  |
| *Fixed Effects* |  |  |  |  |  |  |  |  |  |  |  |  |  |  |  |  |  |
| Intercept |  | 19.34 | 2.14 | <.001 | 12.89 | 2.38 | <.001 | 13.12 | 2.17 | <.001 | 18.46 | 2.18 | <.001 | 83.18 | 1.73 | <.001 |  |
| COVID Onset |  | 1.19 | 1.04 | 0.25 | 4.28 | 1.56 | <.01 | 2.29 | 1.21 | 0.06 | 0.82 | 1.30 | 0.53 | -0.90 | 0.88 | 0.31 |  |
| Gender |  | 0.34 | 3.42 | 0.92 | 3.49 | 4.11 | 0.40 | -0.64 | 2.85 | 0.82 | 1.98 | 3.69 | 0.59 | -2.79 | 2.69 | 0.30 |  |
| Age |  | -0.19 | 0.27 | 0.49 | 0.50 | 0.24 | 0.84 | 0.09 | 0.22 | 0.68 | -0.04 | 0.29 | 0.89 | 0.29 | 0.19 | 0.13 |  |
| MCI |  | -2.73 | 4.12 | 0.51 | -3.48 | 3.13 | 0.27 | -1.91 | 3.09 | 0.54 | -3.32 | 3.87 | 0.39 | 0.88 | 3.34 | 0.80 |  |
| Extraversion |  | -1.29 | 2.51 | 0.61 | -6.26 | 3.24 | 0.06 | -2.46 | 2.30 | 0.29 | -1.30 | 2.51 | 0.61 | 2.81 | 1.70 | 0.10 |  |
| Neuroticism |  | 7.48 | 2.67 | <.01 | -0.07 | 2.67 | 0.98 | 4.56 | 2.40 | 0.06 | 6.55 | 2.70 | 0.02 | -6.53 | 2.11 | <0.01 |  |
| *Random Effects* |  |  |  |  |  |  |  |  |  |  |  |  |  |  |  |  |  |
| Intercept |  | 223.19 | 38.02 | <.001 | 251.57 | 42.48 | <.001 | 143.71 | 24.39 | <.001 | 245.04 | 41.70 | <.001 | 134.24 | 22.86 | <.001 |  |
| COVID Onset |  | 77.36 | 13.58 | <.001 | 186.36 | 30.78 | <.001 | 109.76 | 18.36 | <.001 | 123.48 | 21.12 | <.001 | 56.15 | 9.81 | <.001 |  |
| Residual |  | 211.95 | 2.78 | <.001 | 101.23 | 1.33 | <.001 | 115.58 | 1.52 | <.001 | 229.26 | 3.01 | <.001 | 183.28 | 2.18 | <.001 |  |
| **Models with Personality Interactions** |  |  |  |  |  |  |  |  |  |  |  |  |  |  |  |  |  |
| *Fixed Effects* |  |  |  |  |  |  |  |  |  |  |  |  |  |  |  |  |  |
| Intercept |  | 19.34 | 2.14 | <.001 | 17.74 | 2.40 | <.001 | 13.07 | 2.17 | <.001 | 18.26 | 2.23 | <.001 | 83.31 | 1.76 | <.001 |  |
| COVID Onset |  | 1.23 | 1.33 | 0.35 | 5.41 | 2.12 | 0.01 | 3.03 | 1.64 | 0.07 | 1.42 | 1.71 | 0.41 | -1.23 | 1.17 | 0.29 |  |
| Gender |  | 0.33 | 3.42 | 0.92 | 3.50 | 4.11 | 0.40 | -0.64 | 2.85 | 0.82 | 1.99 | 3.69 | 0.59 | -2.79 | 2.69 | 0.30 |  |
| Age |  | -0.18 | 0.27 | 0.49 | 0.05 | 0.24 | 0.83 | 0.10 | 0.22 | 0.67 | -0.04 | 0.29 | 0.89 | 0.29 | 0.19 | 0.13 |  |
| MCI |  | -2.66 | 4.14 | 0.52 | -2.98 | 3.15 | 0.35 | -1.70 | 3.08 | 0.58 | -2.56 | 3.91 | 0.51 | 0.38 | 3.42 | 0.91 |  |
| COVID Onset X MCI |  | -0.18 | 2.11 | 0.93 | -3.99 | 2.63 | 0.13 | -2.65 | 2.03 | 0.19 | -2.19 | 2.48 | 0.38 | 1.21 | 1.60 | 0.45 |  |
| Extraversion |  | -1.32 | 2.59 | 0.61 | -6.09 | 3.22 | 0.06 | -2.52 | 2.34 | 0.29 | -0.99 | 2.77 | 0.72 | 2.36 | 1.75 | 0.18 |  |
| COVID Onset X Extraversion |  | 0.11 | 1.68 | 0.95 | -1.48 | 1.65 | 0.37 | 0.83 | 1.96 | 0.67 | -0.96 | 2.03 | 0.64 | 1.16 | 1.44 | 0.42 |  |
| Neuroticism |  | 6.84 | 2.60 | 0.01 | -0.26 | 2.65 | 0.92 | 4.33 | 2.40 | 0.07 | 5.52 | 2.69 | 0.04 | -5.89 | 2.12 | <0.01 |  |
| COVID Onset X Neuroticism |  | 2.37 | 1.42 | 0.10 | 1.49 | 1.57 | 0.34 | 3.36 | 1.57 | 0.03 | 3.11 | 1.79 | 0.08 | -1.61 | 1.17 | 0.17 |  |
| *Random Effects* |  |  |  |  |  |  |  |  |  |  |  |  |  |  |  |  |  |
| Intercept |  | 223.28 | 38.04 | <.001 | 251.51 | 42.45 | <.001 | 143.80 | 24.40 | <.001 | 245.03 | 41.69 | <.001 | 134.36 | 22.88 | <.001 |  |
| COVID Onset |  | 78.10 | 13.96 | <.001 | 188.57 | 31.69 | <.001 | 108.61 | 18.56 | <.001 | 122.62 | 21.40 | <.001 | 56.26 | 10.03 | <.001 |  |
| Residual |  | 211.95 | 2.78 | <.001 | 101.23 | 1.33 | <.001 | 115.58 | 1.52 | <.001 | 229.26 | 3.01 | <.001 | 183.28 | 2.18 | <.001 |  |

*Note***.** Results of separate parallel two-level multilevel models are shown. A total of 7,329 observations from 78 participants were collected in 2019 and a total of 7,204 observations from 78 participants were collected in 2020. One participant did not complete the personality measures and thus these 197 observations were excluded from these models. The number of observations used in each model differs because each outcome was assessed at different frequencies. Thought unpleasantness and stress were assessed 6 times per day and thought control, worry, tense/anxious, lonely, depressed, and frustrated were assessed 5 times per day.
